# 

**Published:** 1894-08-04

**Authors:** 


					No. 410'. Vol. XYI.
Saturday,
August 4th, 1894.
WITH EXTRA NURSING SUPPLEMENT.
[Registered at the GeneralPost Office as a Newspaper for Monthly Parts, 6d.; post free, 8d.
transmission in the United Kingdom and Abroad.] Ditto per Annnm, 8s.6d., post free.

				

